# Mothers’ Healthcare Autonomy, Maternal-Health Utilization and Healthcare for Children under-3 Years: Analysis of the Nigeria DHS Data (2008–2018)

**DOI:** 10.3390/ijerph17061816

**Published:** 2020-03-11

**Authors:** Tolulope Ariyo, Quanbao Jiang

**Affiliations:** Institute for Population and Development Studies, School of Public Policy and Administration, Xi’an Jiaotong University, Xi’an 710049, China; ariyotolu@stu.xjtu.edu.cn

**Keywords:** autonomy, maternal health, child healthcare, child morbidity, child immunization, diarrhoea, fever, acute respiratory infection

## Abstract

This study was designed to simultaneously examine if mothers’ personal healthcare autonomy within the household, and the level of their maternal-healthcare utilization, translates into better preventive (complete immunization) and curative (treatments for diarrhoea, fever and acute respiratory infection) efforts on morbidities in child healthcare. We analysed data pooled from three consecutive waves of the Nigeria Demographic and Health Survey: the surveys of 2008, 2013 and 2018. Using a multilevel logistic regression, we estimated the odds ratio for each of the outcome variables while adjusting for covariates. Findings revealed that mothers’ health autonomy is positively associated with child immunization and treatment of morbidities (except diarrhoea), a relationship moderated by the frequency of mothers’ exposure to media. Additionally, mothers’ healthcare utilization is positively associated with complete immunization, and all forms of morbidity treatment (except diarrhoea). Although the relationship between mothers’ healthcare-utilization and child immunization is not dependent on family wealth, however, the relationship between mothers’ healthcare utilization and treatment of morbidity is dependent. Policy effort should be geared towards stimulating mothers to seek appropriate and timely child healthcare and future studies could consider looking into the mediating role of paternal support in this relationship.

## 1. Introduction

In 2018, a global estimate of 5.3 million children under the age of five years died mainly from various forms of curable morbidities, the highest proportion being among the countries of sub-Saharan Africa (SSA). Children in SSA countries were 15 times more susceptible than children in high income countries [1]. In Nigeria, underutilization of Maternal and Child Health (MCH) services accounts for the high rate of both maternal and child mortality [2]. The Nigeria Demographic and Health Survey (NDHS) shows that risk of under-five mortality is higher among children from poor and non-educated families [3]. This is the reality despite the implementation of the National Child Health policy in 2006, aimed at reducing under-five mortality [4], a phenomenon largely accounted for by poor management of sicknesses and diseases [5].

Treatment of childhood illnesses such as diarrhoea, fever and acute respiratory infection (ARI) are usually very effective if the care is sought on time, but a large number of children who are morbid die without ever accessing healthcare facilities [6]. This is partly due to inability to recognize potentially life threatening conditions [6], and the availability of cheaper alternative in traditional medicine which in some cases may not be as effective, especially when diarrhoea, fever and ARI are mere symptoms of other undiagnosed severe illness [7].

Previous studies have shown that caregivers’ decision-making ability and control over economic resources in the household are positively associated with both preventive (immunization) and curative (treatment of illness) efforts on morbidities in children less than five years of age [2,8,9,10,11]. The fulcrum on which this argument rests is that mothers who have the ability to make major decisions within the household may equally have the ability to influence the wellbeing of their children [12,13]. Studies from Nigeria have also suggested that both maternal status (education, age, etc.) and family socio-economic status are predictive of child healthcare [14,15]. However, as all the domains of mothers’ household decision-making autonomy are often assessed as a composite measure, evidence on the specific effect of healthcare autonomy is largely limited. Additionally, an understating of mothers’ disposition to healthcare utilization, determined through the use of maternal and modern healthcare services, could also provide an insight into the relationship between maternal and child healthcare. Therefore, as knowledge is relatively limited regarding this aspect of the relationship of maternal and child healthcare, the aim of this study is to simultaneously examine if mothers’ personal healthcare autonomy within the household, and the level of maternal-healthcare utilization, translate into better preventive and curative efforts on morbidities in child healthcare. To achieve this, we consider the immunization status as a reflection of mothers’ effort towards preventive morbidity, and treatments for diarrhoea, fever and ARI as a reflection of mothers’ effort towards curative morbidity. Findings from this study will be of the utmost importance to health care planners, as well as health professionals, in taking appropriate measures to improve child healthcare utilization.

## 2. Methods

### 2.1. Data Source and Study Design

This study analysed data pooled from three consecutive waves of NDHS spanning 10 years: the surveys of 2008, 2013 and 2018. Nigeria is Africa’s most populous country with an estimated population of over 200 million people of diverse ethnic and cultural backgrounds [16]. The country is divided into six geopolitical zones such that each region is homogenous, sharing similar socio-cultural characteristics. Health-related characteristics such as access to healthcare, environment, housing, etc. are similar within each region [17]. Adult literacy rates for males and females are 71.3% and 52.7%, respectively [18].

The survey was conducted by the National Population Commission (NPC) in collaboration with ICF Macro, Calverton, MD, USA. The sample for the survey was selected using a stratified three-stage cluster design. Firstly, Local Government Areas (LGAs) were selected, then the Primary Sampling Units (PSU,) referred to as clusters, and lastly the selection of households within the selected PSUs. Across the three waves, there were 52,741 households nested within 1400 clusters. Questions were asked relating to household socio-demography, maternal health, the wellbeing of children under-five years old, etc. More information about the survey setting and data collection is provided in final reports from each wave [3,19,20].

### 2.2. Sample Selection Criteria

Among the 33,385, 38,948 and 41,821 women who participated in the 2008, 2013 and 2018 waves, respectively, there were 28,647, 31,482 and 33,924 children born within the five years preceding each of the survey waves. For the purpose of this study, we focused on children who were alive and under-three years, living with their mother, who was married (including cohabitation) and lived with her husband/partner. Respondents who were visitors to the household where they were interviewed were excluded. These, therefore, generated the distribution of observations shown in Table 1. The final number of mother-child dyads included for analysis varied in respect to each outcome variable and the proportion of missing values among all variables of concern. Our analysis was restricted to children less than three years old because, while data that formed part of the components of the independent variables, as well as some for the dependent variables, was captured for children under-five years old in the 2008 and 2013 waves, they were largely restricted to children less than three years old in the 2018 wave.

### 2.3. Outcome Variables

*Immunization status*: We defined a fully immunized child as one between 12 and 23 months old, who received three doses of oral polio vaccine (OPV), three doses of diphtheria, pertussis and tetanus vaccine (DPT), one dose each of Bacille Calmette-Guerin (BCG) and measles vaccine before 12 months of age. During the survey, these were recorded from entries on the child’s vaccination card (where available), or from the account given by the mother (where the card was not available).

*Treatment of diarrhoea:* We calculated this fraction as the number of children under age three who were reported to be sick with diarrhoea two weeks preceding the survey, and who were either taken for treatment at a health facility or given a homemade treatment such as oral rehydration solution (ORS), divided by the number of children under the age of three reported to have had diarrhoea in those two weeks. According to the United Nations International Children’s Emergency Fund (UNICEF), ORS has the capability of treating about 90% of all forms of diarrhoea [21].

*Treatment of fever:* Likewise, we calculated this fraction as the number of children under age three who were reported to be sick with fever two weeks preceding the survey and who were taken for treatment at a health facility, divided by the number of children under the age of three reported to have had fever in those two weeks.

*Treatment of acute respiratory infection (ARI):* In a similar manner, we calculated this fraction as the number of children under age three who were reported to be sick with cough and had difficulty breathing in the two weeks preceding the survey and who were taken for treatment at a health facility, divided by the number of children under the age of three reported to have had cough and difficulty in breathing in those two weeks.

The information about the illnesses and treatment were as reported by the mother. All four outcome variables were binary coded, and consideration for treatment at a health facility was with the exemption of a pharmacy store.

### 2.4. Independent Variables

*Health autonomy:* This variable measures a woman’s ability to make decisions that concern her personal health within the household. This was formed from two question components which had a Cronbach alpha of 0.89. Both questions were collected from the respondents during the survey. One was the question about who makes decisions regarding woman’s healthcare (made either solely or jointly (1), or the decision is someone else’s (0)). The second component was whether getting permission to seek medical help for self at a health facility was a significant problem (0) or not (1). Being items measured on the same scale, both were aggregated. The values which range from minimum of 0 to maximum of 2 were grouped into three ordinal categories: low (0), substantial (1) and high (2).

*Maternal healthcare utilization:* With some modification to the example of Kayode et al. [22], we generated this variable from three components (Cronbach alpha of 0.74) with the aid of principal component analysis (PCA). These components are: (i) attendance at antenatal care at least four times, (ii) receiving anti-tetanus injection, (iii) delivery at a health facility, all during the pregnancy of the child who was part of the dyad selection. Being items measured on different scales, a standardization was performed prior to PCA. This was then grouped into three ordinal categories: low (values below the mean), substantial (values between the mean and 2SD) and high (values above 2SD). This approach was informed by the desire to preserve the ordinal characteristic in respect to relative differences between values.

### 2.5. Covariates

Based on previous studies, we included several covariates at the child, maternal, household and community levels which are likely to be associated with child immunization and treatment of morbidities [7,11,14,23,24,25]. Child variables were: gender, birth order, perceived birth weight and method of delivery. Maternal and household variables were: mother’s age, mother’s education, mother’s frequency of exposure to the media, father’s education and family wealth. Community level variables were: distance to health facility, rural-urban residency and geopolitical zone of residency. Additionally, we controlled for survey year. Religious practice in Nigeria is closely symmetrical to the fault line of geopolitical divisions. The north is predominantly Muslim and the South majorly Christian. Therefore, the inclusion of religion as a variable was no longer considered necessary. Information about distance to medical facility was obtained from the questionnaire during the survey. Respondents were asked to indicate whether the distance required in getting to a medical facility was a significant problem or not when medical help is required. The family wealth variable was constructed using household asset data via a principal components analysis and was already available as part of the DHS dataset. Exposure to the media was related to three questions on the frequency of time the respondent listens to the radio, watches television or reads a newspaper per week.

### 2.6. Statistical Analysis

Descriptive statistics were used to present the characteristics of the study sample. The collinearity diagnostics were checked among predictors and covariates to identify potential issues of multicollinearity. All variance inflation factors (VIF) were below 10, with an average VIF of 1.68.

To take into cognizance the nested nature of the data, (children nested within households, households nested within communities), three level multilevel logistic regression analysis was used to estimate the odds ratio for each of the outcome variables. The 1400 PSUs which served as clusters during the sampling of the DHS survey were taken to represent community levels. We assessed the fixed effect adjusting for observational time as well as covariates. Since we were only interested in the adjusted effect, all covariates were simultaneously introduced into the model. In the random effect, we computed the interclass correlation to examine the within-group variance so as to assess the suitability of our choice of multilevel model. Additionally, we used the log likelihood ratio test to determine goodness of fit [26].

For the purpose of examining potential interactions, we re-grouped our outcome variables into two categories: preventive healthcare (immunization status) and curative healthcare (treatments for fever and ARI). Four cross level interactions were then specified to examine whether the relationship between mothers’ health autonomy and maternal healthcare utilization with preventive and curative healthcare were moderated by mothers’ frequency of exposure to media and family wealth. The basis for this was derived from the Behavioral model of health service utilization [27]. The model suggests that the utilization of healthcare service could be influenced by predisposition to social factors (e.g, education, knowledge) or enabling factors (e.g income). By implication, we expect that the frequency at which mothers are exposed to various sensitizations regarding child health in the media could positively influence health-seeking-behaviour for their child irrespective of the level of their maternal healthcare utilization. Similarly, the financial power available to the family could positively affect the health-seeking-behaviour for their child, irrespective of the health autonomy of the mother.

Furthermore, we conducted a sensitivity analysis. This time we resampled the data by dropping the year 2018 wave, and then expanding the selection criteria to include children under-five years. All analysis was done using STATA 15.1, and results reported at 95% significance threshold.

### 2.7. Ethical Standards Disclosure:

This study was based on secondary analysis of an existing dataset with all participant identifiers removed. The survey instruments followed the Helsinki guidelines and ethical approval was also received from the National Ethics Committee in the Federal Ministry of Health, Abuja, Nigeria, and from the Ethics Committee of the Opinion Research Corporation of Macro International Inc., Calverton, MD, USA (NHREC/01/01/2007). Permission to use the 2018 Nigeria DHS data for this study was obtained from ICF Macro Inc through MEASURE DHS.

## 3. Results

### 3.1. Sample Characteristics

From the aggregated number of observations meeting our selection criteria across the three survey waves in Table 1, 50.7% of the children are males and 49.3% are females. Only 16.4% are first-born children and just 2.0% were delivered through a caesarian section. In relation to maternal characteristics, about 26.5% of mothers are 24 years or less in age, while 23.1% are 35 years and above. 48.5% of mothers had no formal education and about 31.9% had at least a secondary school education. 46.4% of mothers have access to the media at least once per week. With regards to other household factors, 38.4% of fathers had no formal education and 36.8% had a minimum of secondary school education. About 47.1% are from poor and poorest family and 68.2% are residents in rural areas.

### 3.2. Univariate and Bivariate Analysis

**Figure A1:**
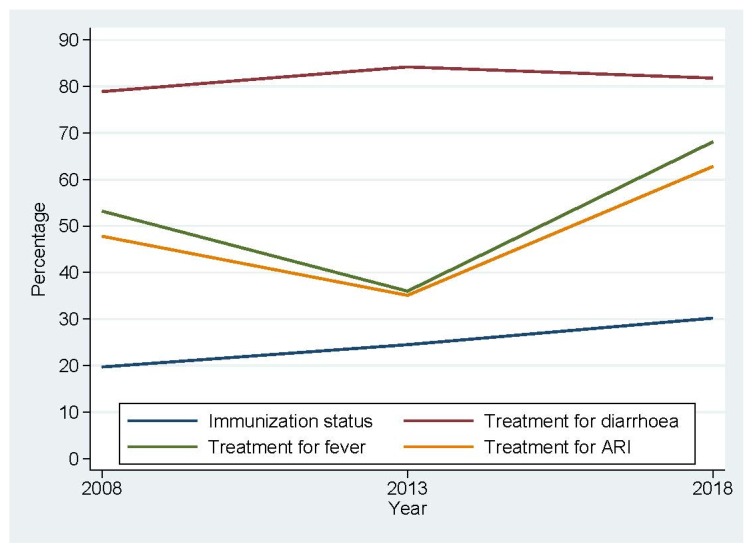
The trends of immunization status and treatment for morbidities among children less than three years | ARI = Acute respiratory infection.

Across the four samples shown in Table 2, between 88% and 89% of mothers had substantial or high level of health autonomy, while the proportion was no more than 44.2% for maternal healthcare utilization. Both immunization status and treatment for diarrhoea progressed between 2008 and 2018. Although, treatment for fever and ARI also improved during this period, there was a decline in 2013 (See Appendix A Figure A1). From the aggregated data, 23.8% of children were fully immunized and 81.9% of children who were reported to have had diarrhoea received treatment. In addition, only 51.5% of children reported to have had fever received medical treatment, and just 46.4% of children reported to have had ARI and difficulty in breathing received medical treatment.

Furthermore, there was a significant bivariate association between mother’s health autonomy, maternal healthcare utilization and each of the four child healthcare variables of concern (χ^2^ *p*-value < 0.01).

### 3.3. Regression Result

The results of the null models showed significant variation for each of the outcome variables. A variability of up to 86%, 61%, 83% and 87% were associated with immunization and treatments for diarrhoea, fever and ARI at both the community and household levels, respectively (Table 3). This, therefore, provides a justification for the choice of multilevel model over ordinary logistic regression.

#### 3.3.1. Association between Mother’s Health Autonomy, Healthcare Utilization and Child’s Immunization

Compared to the survey of 2008, the two subsequent surveys showed higher likelihood for complete child immunization and treatment of morbidities, except in 2018 when children were almost just as likely to be treated for diarrhoea (Table 4). Furthermore, compared to children whose mothers have low healthcare autonomy, those whose mothers have substantial and high healthcare autonomy were 53% (AOR = 1.53; CI = 1.12–2.11; *p* < 0.01) and almost twice more than likely (AOR = 1.87; CI = 1.34–2.62; *p* < 0.01) to be fully immunized, respectively. Similarly, children whose mothers have substantial and high healthcare utilization were more than three times as likely (AOR = 3.53; CI = 2.82–4.41; *p* < 0.01) and more than five times as likely (AOR = 5.61; CI = 4.01–7.84; *p* < 0.01) to be fully immunized compared to children whose mothers have low maternal healthcare utilization.

#### 3.3.2. Association between Mother’s Health Autonomy, Healthcare Utilization and Treatment of Diarrhoea

Compared to children whose mothers have low healthcare autonomy, those whose mothers have substantial and high healthcare autonomy were almost as likely (AOR = 1.08; CI = 0.82–1.43; *p* = 0.1) and 16% more likely (AOR = 1.16; CI = 0.83–1.62; *p* = 0.1) to be treated for diarrhea, respectively. But these were not statically significant.

Children whose mothers have substantial and high maternal healthcare utilization were 25% (AOR = 1.25; CI = 0.96–1.61; *p* = 0.1) and 46% (AOR = 1.46; CI = 0.81–2.63; *p* = 0.1) more likely to be treated for diarrhea, respectively. However, these effects were not statistically significant at 95%.

#### 3.3.3. Association between Mother’s Health Autonomy, Healthcare Utilization and Treatment of Fever

Compared to children whose mothers have low health autonomy, those whose mothers have substantial and high autonomy were 53% (AOR = 1.53; CI = 1.15–2.05; *p* < 0.01) and 51% (AOR = 1.51; CI = 1.09–2.10; *p* < 0.01) more likely to be treated for fever, respectively. Furthermore, in regards to maternal healthcare utilization, children whose mothers have substantial and high healthcare utilization were nearly twice as likely (AOR = 1.89; CI = 1.47–2.43; *p* < 0.01) and more than twice as likely (AOR = 2.21; CI = 1.43–3.41; *p* < 0.01) to receive medical treatment for reported cases of fever.

#### 3.3.4. Association between Mother’s Health Autonomy, Healthcare Utilization and Treatment of ARI

Compared to children whose mothers have low health autonomy, those whose mothers have substantial and high autonomy were 65% (AOR = 1.65; CI = 1.09–2.52; *p* < 0.05) and 24% (AOR = 1.24; CI = 0.78–1.99; *p* = 0.1) more likely to be treated for ARI, respectively. However, the effect for the high autonomy group was not statistically significant. On the other hand, compared to children whose mothers have low maternal healthcare utilization, those whose mothers have substantial and high maternal healthcare utilization were 63% (AOR = 1.63; CI = 1.21–2.21; *p* < 0.01) and more than twice as likely (AOR = 2.67; CI = 1.45–4.91; *p* < 0.01) to be treated for ARI, respectively. The result from the sensitivity test was similar in direction and magnitude of association (See Table A1 and Table A2)

#### 3.3.5. Interactions Effect

**Figure 1:**
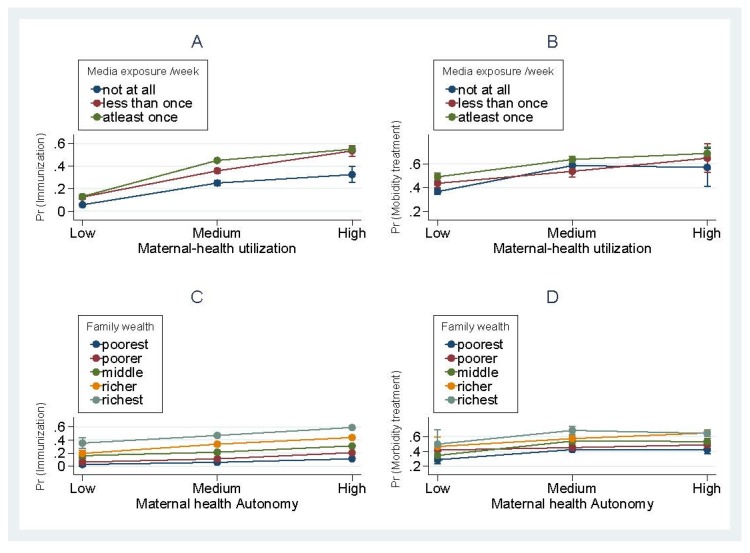
The relationship between maternal-health utilization and child healthcare as moderated by mothers’ exposure to media and the relationship between health autonomy and child healthcare as moderated by family wealth.

As shown in Figure 1, the relationship between maternal healthcare utilization and child immunization was slightly moderated by mothers’ frequency of exposure to the media (Panel A). However, there was a stronger moderation pattern in the relationship between mothers’ healthcare utilization and treatment of morbidities (Panel B). While the relationship between mothers’ health autonomy and child immunization was not moderated by family wealth (Panel C), the relationship between mothers’ health autonomy and treatment of morbidity was so influenced (Panel D).

## 4. Discussion

### 4.1. Summary of Results

In this study we used pooled data from three consecutive waves of NDHS which covered a 10 year period. Using a multilevel logistic regression, we simultaneously examined the associative effect of mothers’ health autonomy and healthcare utilization on preventive child healthcare (immunization status) and three forms of curative child healthcare (treatments of diarrhoea, fever and acute respiratory infection) while adjusting for other covariates at various levels. Our findings revealed that mothers’ health autonomy is positively associated with preventive child healthcare. Furthermore, mothers’ health autonomy is associated with curative child healthcare (except for diarrhoea). However, these relationships are moderated by the frequency of mothers’ exposure to media such that the effect is stronger when exposure to media is high. Additionally, mothers’ healthcare utilization is positively associated with both preventive child healthcare and all forms of curative child healthcare (except for diarrhoea). While the effect of the relationship between mothers’ healthcare utilization and child immunization is not dependent on family wealth, the effect of the relationship between mothers’ healthcare utilization and treatment of morbidity is moderated by family wealth such that, when family wealth is low, the effect is weakened.

The current study contributes to knowledge by showing how mothers’ health autonomy (among other domains of mothers’ decision-making autonomy) and mothers’ healthcare utilization relates to both preventive and curative child healthcare. We also showed how these relationships are moderated by both mother’s exposure to the media and family wealth.

### 4.2. Relevance of Result

Relationship between Maternal Healthcare Autonomy and Child Healthcare.

Our findings complements other studies from Nigeria [2,7,11,28], other SSA countries [6,9,23] and Asian countries [8,10,29], which have reported several agencies of mothers’ status to be associated with health-seeking-behaviour for children.

In our study, we observed that mothers’ health autonomy showed an improved trend across the years of survey (data not shown). Likewise, the trends for child immunization and treatment of morbidities also showed an improvement. However, the overall percentage was far below international recommendations. Cultural practices in Nigeria are largely patriarchal, meaning few women may have a say regarding their own health [2]. However, women who have the ability to decide on their own healthcare may equally be in position to do same for their children, seeking appropriate healthcare for them when such is required [12]. With regards to ARI, while there was a significant relationship with the substantial health autonomy group, it was shocking to observe that there was no significant relationship for the high health autonomy group. The reason for this is unknown.

The relationship between mother’s health autonomy and treatment for diarrhoea was found not to be statistically significant. This could be explained by the fact that there are effective homemade treatments for diarrhoea such as ORS. The efficacy of this cost effective solution which only requires salt, sugar and clean water may have made it unnecessary to seek further medical care for this sickness, as was also observed in a study from southern Malawi [30]. Additionally, a study conducted in the northern part of Nigeria revealed that people who participated in an ORS knowledge programme demonstrated better skills for managing diarrhoea at home than people who did not participate [31].

For the two other forms of morbidities (fever and ARI), their association was dependent on family wealth. Even when mothers have adequate health autonomy to make health decisions for themselves or their children, the lack of an enabling factor (money to foot medical bills) may be an obstacle. On the other hand, the reason wealth does not moderate the relationship between health autonomy and child immunization could be subject to the fact that immunization is free across most government owned health facilities.

### 4.3. Relationship between Maternal Healthcare Utilization and Child Healthcare

There appeared to be no observable improvement pertaining to mothers’ healthcare utilization between 2008 and 2013. However, there was a relative improvement in the year 2018 survey (data not shown). In our study, maternal healthcare utilization was associated with immunization status in children as well as treatments of all morbidities (except diarrhea, probably for the same reason stated above). Mothers who are disposed to utilizing modern healthcare services will be likely to do the same for their children. This relationship was moderated by mothers’ frequency of exposure to the media. According to the behavioural health service utilization model proposed by Andersen [27], education (knowledge) is a crucial factor in the utilization of health services. Sensitization to the importance of timely and appropriate child healthcare in the media could positively influence the utilization of healthcare service for both preventive and curative child healthcare. While we admit that maternal level of education may also play a similar moderating role, the most important factor is having knowledge and awareness about appropriate child healthcare in relation to identification of symptoms of illnesses. Such information is more likely to be obtained in an updated form through public sensitization.

Furthermore, health literacy is regarded as an important concept in healthcare utilization. As defined by Ratzan and Parker (2000), and adopted by the Institute of Medicine, health literacy relates to the ability of individuals to obtain, process and understand health messages so as to make informed health decisions [32]. However, as components that define this variable are not directly measured in the DHS surveys, other variables such as education and exposure to media have been considered as proxies in some studies [33]. In our study, mothers’ high exposure to the media was related to better child health (except diarrhoea), and increased level of mothers’ education was associated with better healthcare with regards to immunization and treatment for fever.

### 4.4. Policy Implication

Findings from this study reveal that the government policy on free child immunization has created a phenomenon where socioeconomic status is no longer a barrier for a new-born child getting all vaccines required before the age of one. However, much greater awareness is still needed in regard to the importance of full immunization as the rate is far below the 90% internationally recommended target. Additionally, as treatment of morbidities was dependent on wealth, easily accessible and free universal MCH service is of the utmost importance. Evidence suggests that there was relative increase in the demand and utilization of MCH services between 2009 and 2016, when the National Health Insurance Scheme implemented a free MCH programme across 12 states in Nigeria. However, issues of governance, alleged corrupt practices and problem of funding were reasons identified for its discontinuation [34].

### 4.5. Strength and Limitations of the Study

The strength of our study lies in the use of a large and representative dataset that makes it possible to generalize our findings. We also used an appropriate statistical method that took into cognizance the clustered characteristics associated with the data. However, several limitations are associated with this study. Firstly, the use of cross-sectional data, as with similar study designs, prevents the establishment of a causal relationship. Secondly, due to the fact that data on some of the variables of concern were largely restricted to children under-3 years of age in the 2018 wave, analysis was restricted to children within this age group. Furthermore, as no medical diagnosis was relied upon during the collection of data regarding cases of morbidities, over or under reporting is probable as some mothers might not have been able to accurately identify some symptoms of these morbidities. Lastly, respondents’ level of health literacy is an important concept in the framework of healthcare utilization. However, as this variable was not captured during the survey, we had to rely on proxies.

## 5. Conclusions

Findings from this study show that mothers’ healthcare autonomy is positively associated with complete immunization as well as treatment of fever and ARI in children under the age of three years, but the relationship in the latter is dependent on household wealth. Additionally, mothers’ healthcare utilization, which was determined through the utilization of maternal and modern healthcare services, is similarly associated with complete immunization and the treatment of fever and ARI in children less than three years of age, a relationship which is however moderated by the frequency of mothers’ exposure to media. While the sustainability of the current policy of free immunization is of necessity, health policies that could reduce the burden of the cost of healthcare service are imperative. Effort should also be geared towards stimulating mothers on the need for seeking appropriate and timely child healthcare. Future studies could consider looking into the mediating role of paternal support in this relationship.

## Figures and Tables

**Table 1:** Distribution of Observation Meeting Main Selection Criteria, Followed up by Year of Survey.

	Total		Year			
			2008	2013	2018	
	N =	%	%	%	%	*p*-Value
Gender						0.803
Male	17176	50.7	50.2	50.8	51.5	
Female	16693	49.3	49.8	49.2	48.5	
Birth order						0.001
First born	5541	16.4	16.2	16.6	16.2	
2–3rd	11162	33.0	32.9	32.7	33.8	
4–6th	11234	33.2	33.0	32.9	34.3	
7th+	5932	17.5	18.0	17.8	15.7	
Birth weight						0.000
Small	5003	14.9	15.5	15.0	13.5	
Average	13996	41.8	37.9	40.7	52.6	
Large	14523	43.3	46.6	44.3	33.9	
Method of delivery						0.000
Normal	33046	98.0	98.5	97.9	97.0	
Caesarean session	687	2.0	1.6	2.1	3.0	
Mother’s Age						0.000
15–19	2044	6.0	6.9	5.8	4.9	
20–24	6912	20.4	20.7	20.6	19.2	
25–29	9770	28.9	29.1	28.4	29.4	
30–34	7319	21.6	20.7	21.8	23.2	
35+	7824	23.1	22.7	23.4	23.3	
Mother’s Education						0.000
no education	16420	48.5	52.0	48.5	41.0	
primary	6649	19.6	21.9	19.3	15.6	
secondary	8624	25.5	21.3	25.7	33.9	
higher	2176	6.4	4.9	6.5	9.5	
Mother’s EM/Week						0.000
None	11784	34.8	34.6	35.0	35.1	
less than once	6359	18.8	13.4	21.8	23.2	
at least once	15700	46.4	52.1	43.2	41.8	
Father’s Education						0.000
no education	12854	38.4	41.9	38.2	31.3	
primary	6407	19.1	21.3	19.3	14.2	
secondary	9948	29.7	26.2	29.2	38.6	
higher	4275	12.8	10.6	13.4	15.9	
Family wealth						0.000
poorest	8251	24.4	27.1	23.3	21.1	
poor	7685	22.7	23.6	23.0	19.9	
middle	6433	19.0	18.2	18.8	21.2	
richer	6076	17.9	16.7	18.1	20.2	
richest	5424	16.0	14.4	16.8	17.6	
DMF						0.000
Big problem	11759	34.8	40.2	32.4	29.1	
not a big problem	21997	65.2	59.8	67.6	71.0	
Residency						0.000
Urban	10781	31.8	27.7	32.9	38.2	
Rural	23088	68.2	72.3	67.2	61.8	
Geopolitical zone						0.000
Northcentral	5533	16.3	17.3	15.1	17.1	
Northeast	7481	22.1	23.9	21.6	19.4	
Northwest	10192	30.1	28.7	32.8	26.5	
Southeast	2962	8.8	7.9	7.8	12.9	
Southsouth	3530	10.4	10.2	10.8	10.1	
Southwest	4171	12.3	11.9	12.0	13.9	

1. *p*-value = Kruskal-Wallis test for difference among samples of survey years, α = 0.05; 2. Mother’s EM = Mother’s exposure to media; 3. DMF = Distance to medical facility.

**Table 2:** Aggregated Cross Tabulation of Independent Variables by Dependent Variables (2008–2018).

	**Immunization N = 16694**							**Diarrhoea N = 4047**						
	**No**	**%**	Yes	**%**	**Total**	**%**	**χ2 *P*-Value**	**No**	**%**	**Yes**	**%**	**Total**	**%**	**χ2 *P*-Value**
Health autonomy							<0.01							<0.01
Low	1674	13.1	145	3.7	1819	10.9		113	15.5	373	11.3	486	12.0	
Substantial	7507	59.0	1739	43.9	9246	55.4		475	65.0	2022	60.3	2497	61.7	
High	3548	27.9	2081	52.5	5629	33.7		143	19.6	921	27.8	1064	26.3	
Health utilization							<0.01							<0.01
Low	8464	66.5	848	21.4	9312	55.8		561	76.7	1999	60.3	2560	63.3	
Substantial	3555	27.9	2309	58.2	5864	35.1		150	20.5	1112	33.5	1262	31.2	
High	710	5.6	808	20.4	1518	9.1		20	2.7	205	6.2	225	5.6	
	**Fever N = 4973**							**ARI N = 1575**						
	**No**	**%**	**Yes**	**%**	**Total**	**%**	**χ2 *P*-Value**	**No**	**%**	**Yes**	**%**	**Total**	**%**	**χ2 *P*-Value**
Health autonomy							<0.01							<0.01
Low	360	14.9	216	8.4	576	11.6		122	14.5	53	7.3	175	11.1	
Substantial	1416	58.7	1483	58.0	2899	58.3		481	57.0	440	60.2	921	58.5	
High	638	26.4	860	33.6	1498	30.1		241	28.6	238	32.6	479	30.5	
Health utilization							<0.01							<0.01
Low	1653	68.5	1263	49.4	2916	58.6		600	71.1	345	47.2	945	60.0	
Substantial	643	26.6	1058	41.3	1701	34.2		216	25.6	321	43.9	537	34.1	
High	118	4.9	238	9.3	356	7.2		28	3.3	65	8.9	93	5.9	

1. N = Number of observations; 2. ARI = Acute respiratory infection; 3. Health Autonomy. Low (respondent could not decide on personal healthcare and, at the same time, had a significant problem seeking permission to go for medical help at a health facility). Substantial (respondent could either decide on personal healthcare OR had no significant problem about seeking permission to go for medical help at a health facility); High (respondent could decide on personal healthcare and also had no significant problem about seeking permission to go for medical help at a health facility). Healthcare Utilization: (generated from principal component analysis (PCA)). Low (values below the mean); Substantial (values between the mean and 2SD). High (values above 2SD).

**Table 3:** Random Effects from the Multilevel Logistic Regression on Preventive and Curative Child Healthcare in Nigeria. (2008–2018).

RANDOM EFFECT	Immunization		Diarrhoea		Fever		ARI	
		CI		CI		CI		CI
Variance at community level	0.00 ***	(0.00–0.01)	2.85 ***	(1.34–6.07)	0.10 ***	(0.05–0.22)	0.13 ***	(0.05–0.32)
Variance at Household	2.46 ***	(1.82–3.32)	1.30 **	(1.04–1.63)	1.57 ***	(1.19–2.06)	1.67 ***	(1.15–2.43)
Log likelihood	−6880.2246		−1788.5715		−3065.34		−934.90057	
Model fit Statistics LR test	272.25 ***		11.49***		43.13 ***		14.52 ***	
ICC from Null model:								
Community	0.25	(0.23–0.27)	0.11	(0.06–0.16)	0.13	(0.10–0.18)	0.21	(0.05–0.22)
Household | Community	0.86	(0.83–0.88)	0.61	(0.44–0.76)	0.83	(0.74–0.90)	0.87	(0.05–0.22)
Observations	16694		4047		4973		1575	
Number of groups	1295		892		1037		665	

CI = Confidence Interval; ICC = Inter class correlation; *** *p*<0.01, ** *p*<0.05; ARI = Acute Respiratory Infection.

**Table 4:** Adjusted Odds Ratio from Multilevel Logistic Regression on Preventive and Curative Child Healthcare in Nigeria (2008–2018).

Variables	Immunization		Diarrhoea		Fever		ARI	
	AOR	CI	AOR	CI	AOR	CI	AOR	CI
Survey year								
ref = 2008								
2013	1.63 ***	(1.36–1.95)	1.42 ***	(1.12–1.78)	0.32 ***	(0.24–0.44)	0.44 ***	(0.33–0.60)
2018	2.33 ***	(1.84–2.94)	1.06	(0.82–1.37)	2.43 ***	(1.82–3.25)	1.98 ***	(1.41–2.77)
Health Autonomy								
ref = Low								
Medium	1.53 ***	(1.12–2.11)	1.08	(0.82–1.43)	1.53 ***	(1.15–2.05)	1.65 **	(1.09–2.52)
High	1.87 ***	(1.34–2.62)	1.16	(0.83–1.62)	1.51 **	(1.09–2.10)	1.24	(0.78–1.99)
Health Utilization								
ref = Low								
Medium	3.53 ***	(2.82–4.41)	1.25 *	(0.96–1.61)	1.89 ***	(1.47–2.43)	1.63 ***	(1.21–2.21)
High	5.61 ***	(4.01–7.84)	1.46	(0.81–2.63)	2.21 ***	(1.43–3.41)	2.67 ***	(1.45–4.91)
**Child Level Covariates**								
Gender								
ref = Male								
Female	1.02	(0.89–1.18)	0.91	(0.76–1.10)	0.99	(0.84–1.16)	0.97	(0.76–1.23)
Birth order								
ref = First born								
2–3rd	0.72 ***	(0.57–0.92)	1.11	(0.80–1.54)	0.88	(0.66–1.18)	0.97	(0.64–1.47)
4–6th	0.55 ***	(0.42–0.73)	1.15	(0.79–1.67)	0.90	(0.65–1.25)	0.78	(0.48–1.27)
7th+	0.52 ***	(0.36–0.74)	1.45	(0.90–2.33)	0.93	(0.62–1.39)	0.80	(0.44–1.44)
PWB								
ref = Small								
Average	1.37 **	(1.08–1.74)	0.96	(0.75–1.22)	1.31 **	(1.02–1.67)	1.51 **	(1.07–2.13)
Large	1.29 **	(1.01–1.64)	1.26 *	(0.98–1.63)	1.57 ***	(1.22–2.04)	2.17 ***	(1.54–3.08)
Delivery method								
Ref = normal birth								
C & S	1.84 ***	(1.18–2.86)	0.55	(0.22–1.42)	0.96	(0.50–1.86)	0.95	(0.36–2.55)
**Maternal and Household Level Covariates**								
Mothers’ age								
Ref = 15–19 years								
20–24	1.70 **	(1.08–2.68)	0.83	(0.55–1.23)	1.52 **	(1.01–2.29)	1.58	(0.89–2.80)
25–29	2.75 ***	(1.72–4.40)	0.96	(0.62–1.48)	1.41	(0.92–2.17)	1.50	(0.82–2.75)
30–34	3.17 ***	(1.92–5.21)	1.13	(0.69–1.85)	1.56 *	(0.98–2.50)	1.78 *	(0.92–3.44)
35+	3.55 ***	(2.11–5.97)	1.07	(0.63–1.82)	1.48	(0.90–2.43)	1.77	(0.88–3.56)
Mothers’ education								
ref= No education								
primary	1.87 ***	(1.47–2.37)	1.04	(0.78–1.38)	1.57 ***	(1.19–2.07)	1.29	(0.90–1.85)
secondary	2.89 ***	(2.19–3.80)	1.10	(0.75–1.63)	1.58 ***	(1.13–2.20)	1.42	(0.92–2.20)
higher	2.78 ***	(1.89–4.08)	1.18	(0.52–2.71)	2.68 ***	(1.50–4.79)	1.63	(0.74–3.59)
Media exposure/Week								
ref = None								
less than once	1.29 **	(1.02–1.63)	1.09	(0.84–1.43)	1.04	(0.81–1.33)	1.27	(0.87–1.85)
at least once	1.51 ***	(1.22–1.87)	1.25 *	(0.98–1.60)	1.46 ***	(1.16–1.85)	1.69 ***	(1.24–2.30)
Father’s education								
ref = low								
primary	1.86 ***	(1.44–2.42)	1.33 **	(1.00–1.76)	1.11	(0.86–1.44)	0.78	(0.53–1.15)
secondary	1.98 ***	(1.52–2.56)	1.49 **	(1.08–2.06)	1.21	(0.92–1.60)	0.94	(0.64–1.38)
higher	2.57 ***	(1.87–3.54)	1.62 **	(1.00–2.63)	1.34	(0.92–1.94)	2.05 ***	(1.22–3.43)
Family wealth								
ref = poorest								
poor	1.43 **	(1.08–1.88)	1.23 *	(0.97–1.56)	1.34 **	(1.05–1.71)	0.98	(0.69–1.39)
middle	1.89 ***	(1.41–2.53)	1.91 ***	(1.34–2.72)	1.30 *	(0.98–1.75)	0.87	(0.58–1.31)
richer	2.62 ***	(1.88–3.67)	1.28	(0.83–1.98)	1.83 ***	(1.26–2.68)	1.17	(0.70–1.98)
richest	4.02 ***	(2.69–6.02)	1.91 **	(1.01–3.60)	1.91 ***	(1.19–3.08)	1.19	(0.60–2.36)
**Community Level Covariates**								
Residency								
ref = Urban								
Rural	0.85	(0.70–1.04)	0.81	(0.59–1.10)	1.40 **	(1.08–1.80)	1.21	(0.84–1.74)
Geopolitical zone								
ref = Northcentral								
North east	0.57 ***	(0.43–0.75)	0.82	(0.57–1.17)	1.14	(0.84–1.55)	0.74	(0.49–1.11)
Northwest	0.28 ***	(0.21–0.38)	0.74	(0.50–1.07)	1.47 **	(1.06–2.05)	1.00	(0.62–1.64)
Southeast	1.15	(0.84–1.57)	1.80	(0.86–3.77)	0.84	(0.56–1.26)	0.97	(0.54–1.72)
Southsouth	1.20	(0.90–1.62)	0.68	(0.36–1.29)	0.71 *	(0.47–1.05)	1.10	(0.63–1.90)
Southwest	0.54 ***	(0.40–0.73)	0.60*	(0.34–1.04)	0.66 *	(0.41–1.05)	0.52	(0.24–1.14)
Distance to medical facility								
(ref = Big problem)								
Not a problem	1.21 **	(1.02–1.45)	1.28 **	(1.04–1.59)	1.31 ***	(1.08–1.59)	1.58 ***	(1.21–2.08)
**Constant (Intercept)**	143.29 ***	(22.82–99.51)	1.56	(0.38–6.46)	5.99 *	(0.94–38.08)	1.00	(1.00–1.00)
Observations	16694		4047		4973		1575	
Number of groups	1295		892		1037		665	

AOR = Adjusted odds ratio; CI = Confidence Interval; ref = Reference group; C&S = Caesarean session; PWB = Perceived Birth Weight; *** *p* < 0.01, ** *p* < 0.05, * *p* < 0.1; ARI = Acute Respiratory Infection.

## Data Availability

The dataset used for analysis and reaching the conclusions of this study is available online at MEASURE DHS (https://www.dhsprogram.com/data/available-datasets.cfm). They are released upon request subject to approval.

## Appendix A

**Table A1:** Adjusted odds ratio from multilevel logistic regression on preventive and curative infant and child health in Nigeria (2008–2018).

Variables	Immunization±		Diarrhoea		Fever		ARI	
	AOR	CI	AOR	CI	AOR	CI	AOR	CI
Health Autonomy (B = Low)								
substantial	1.53 ***	(1.12–2.11)	1.08	(0.82–1.43)	1.53 ***	(1.15–2.05)	1.65 **	(1.09–2.52)
High	1.87 ***	(1.34–2.62)	1.16	(0.83–1.62)	1.51 **	(1.09–2.10)	1.24	(0.78–1.99)
Health Utilization (B = Low)								
Medium	3.53 ***	(2.82–4.41)	1.25 *	(0.96–1.61)	1.89 ***	(1.47–2.43)	1.63 ***	(1.21–2.21)
High	5.61 ***	(4.01–7.84)	1.46	(0.81–2.63)	2.21 ***	(1.43–3.41)	2.67 ***	(1.45–4.91)
Observations	16694		4047		4973		1575	
Number of groups	1295		892		1037		665	

*** *p*< 0.01, ** *p* < 0.05, * *p* < 0.1

**Table A2:** Adjusted odds ratio of robustness check from multilevel logistic regression on preventive and curative infant and child health in Nigeria (2008–2013).

	Immunization±		Diarrhoea		Fever		ARI	
	AOR	CI	AOR	CI	AOR	CI	AOR	CI
Health Autonomy(B = Low)								
substantial	1.49 ***	(1.11–2.01)	1.19	(0.87–1.62)	1.47 **	(1.06–2.04)	1.56 *	(0.93–2.62)
High	1.64 ***	(1.20–2.24)	1.04	(0.71–1.50)	1.28	(0.89–1.85)	1.13	(0.65–1.97)
Health Utilization (B = Low)								
Medium	3.65 ***	(2.95–4.51)	1.24	(0.92–1.68)	2.03 ***	(1.52–2.70)	2.11 ***	(1.33–3.34)
High	5.33 ***	(3.96–7.17)	1.59	(0.80–3.17)	3.64 ***	(2.14–6.20)	5.07 ***	(1.97–13.06)
Observations	18119		3535		4151		1342	
Number of groups	904		728		841		537	

Table A1 = children < 36 months old; Table A2 = children < = 59 months old; ± = Children > = 12 months old; B = Reference Group; *** *p*< 0.01, ** *p* < 0.05, * *p* < 0.1; Control variables: Child level: Gender, birth order, perceived birth weight, method of birth; Maternal and household level: Mother’s age, mother’s education, mother media exposure, father’s education, wealth; Community level: Distance to medical facility, residency, geopolitical zone; Others: Survey year.
